# Postexercise Cooling Lowers Skeletal Muscle Microvascular Perfusion and Blunts Amino Acid Incorporation into Muscle Tissue in Active Young Adults

**DOI:** 10.1249/MSS.0000000000003723

**Published:** 2025-04-18

**Authors:** MILAN W. BETZ, CAS J. FUCHS, FINLAY CHEDD, ALEJANDRA P. MONSEGUE, FLORIS K. HENDRIKS, JANNEAU M. X. VAN KRANENBURG, JOY GOESSENS, ALFONS J. H. M. HOUBEN, LEX B. VERDIJK, LUC J. C. VAN LOON, TIM SNIJDERS

**Affiliations:** 1 Department of Human Biology, NUTRIM Institute of Nutrition and Translational Research in Metabolism, Maastricht University Medical Centre+, Maastricht, THE NETHERLANDS; 2 Department of Internal Medicine, Cardiovascular Research Institute Maastricht (CARIM), Maastricht University, Maastricht, THE NETHERLANDS

**Keywords:** BLOOD FLOW, CEUS, CEU, CWI, CRYOTHERAPY, MICROCIRCULATION

## Abstract

**Purpose:**

Cold-water immersion lowers muscle protein synthesis rates during postexercise recovery. Whether this effect can be explained by lower muscle microvascular perfusion and a subsequent decline in postprandial amino acid incorporation into muscle tissue after cooling is currently unknown.

**Methods:**

Twelve young males (24 ± 4 yr) performed a single resistance exercise session followed by water immersion for 20 min with one leg immersed in cold water (8°C: COLD) and the contralateral leg in thermoneutral water (30°C: CON). After immersion, a beverage was ingested containing 20 g free amino acids, 0.25 g L-[ring-^13^C_6_]-phenylalanine, and 45 g carbohydrates. Microvascular perfusion of the *vastus lateralis* muscle was assessed for both legs using contrast-enhanced ultrasound at rest, immediately after exercise and water immersion, and at *t* = 60 and *t* = 180 min after beverage ingestion. A muscle biopsy sample (*vastus lateralis*) was collected from both legs (*t* = 240 min) to determine amino acid tracer incorporation.

**Results:**

Microvascular blood volume was significantly lower in the COLD versus CON leg immediately after water immersion (1.24 ± 0.82 vs 3.13 ± 1.64 video intensity, respectively, *P* < 0.001) and remained lower at *t* = 60 and *t* = 180 min after beverage ingestion (0.90 ± 0.84 vs 1.53 ± 0.98, and 2.10 ± 2.53 vs 2.77 ± 2.81 video intensity, respectively, both *P* < 0.05). Exogenous amino acid incorporation into muscle protein was lower in the COLD versus CON leg (0.011 ± 0.004 vs 0.016 ± 0.005 mole percent excess, respectively, *P* < 0.001). The difference in postprandial amino acid incorporation into muscle protein between the COLD and the CON legs was strongly associated with the difference in microvascular blood volume between the two legs during recovery (*r* = 0.65, *P* < 0.05).

**Conclusions:**

Cold-water immersion during postexercise recovery greatly reduces muscle microvascular perfusion and blunts postprandial amino acid incorporation in muscle.

Cold-water immersion is commonly applied by athletes to facilitate postexercise muscle recovery (1). Postexercise cold-water immersion has been reported to reduce delayed onset muscle soreness (2–4), muscle swelling (3,5–7), and improve muscle function/performance recovery (3,7–9). These proposed benefits of cold-water immersion may be largely analgesic and seem at least partly based on subjective outcome measures (4). In fact, recent studies suggest that postexercise cooling may even impede postexercise muscle recovery and, as such, compromise long-term muscle adaptive responses (10,11).

Cold-water immersion has been shown to significantly reduce muscle temperature, leading to a substantial (~55%–75%) reduction in lower limb arterial blood flow, typically assessed by Doppler ultrasound (12,13). Using positron emission tomography (PET) and [^15^O]H_2_O, Mawhinney et al. (14) similarly showed that *vastus lateralis* perfusion is markedly diminished under this condition. The reduction in postexercise blood flow is hypothesized to be a key mechanism underlying the observed impairments in long-term muscle adaptation to exercise training (11,15). However, blood flow measurements by Doppler ultrasound and/or PET [^15^O]H_2_O only provide information on blood supply toward the muscle or whole muscle perfusion but do not specifically assess muscle microvascular perfusion *per se*. The microvasculature is particularly important, as it is the site where oxygen, nutrients, and growth factors exchange with muscle fibers (16). Contrast-enhanced ultrasound (CEUS) is a technique to assess tissue perfusion dynamics and can be applied effectively to study skeletal muscle microvascular perfusion *in vivo* in humans (17–22). However, the impact of postexercise cold-water immersion on muscle microvascular perfusion, as measured by CEUS, is currently unknown.

Dietary protein ingestion is essential to support postexercise muscle recovery and adaptation (23–27). After digestion, dietary protein derived amino acids are absorbed, released into the circulation, and subsequently transported to peripheral tissues, including muscle (28). The muscle microvasculature (i.e., the muscle fiber capillary network) is where the amino acids are taken up by the muscle fibers to support postexercise muscle reconditioning. Previously, our laboratory has shown that postexercise cold-water immersion blunts the increase in muscle protein synthesis rates after protein ingestion in healthy young males (11). Whether this effect can be explained by a cold-water immersion-induced reduction in muscle microvascular perfusion and a concomitant decline in postprandial amino acid incorporation into muscle tissue remains unknown. Therefore, in this study, we determined the impact of postexercise cold-water immersion on muscle microvascular perfusion, assessed by CEUS, in recreationally active young adult males. In addition, we assessed whether any cold-water immersion-induced change in muscle microvascular perfusion might be related to the capacity for postprandial exogenous amino acid incorporation into muscle tissue during postexercise recovery.

## METHODS

### Ethical approval

This study was approved by the Medical Ethics Committee of the Maastricht University Medical Centre+/Maastricht University (METC 21-049) and conformed to the principles outlined in the Declaration of Helsinki for use of human subjects and tissue. Participants were fully informed of the nature and possible risks of the experimental procedures before their written informed consent was obtained. This study was registered at the International Clinical Trials Registry Platform (https://trialsearch.who.int) as NL9811 and was independently monitored by the Clinical Trial Center Maastricht.

### Participants

Twelve healthy young men (age 24 ± 4 yr) participated in this randomized controlled within-subject (i.e., unilateral model) designed study between May 2022 and March 2023 (see Supplemental Fig. 1, http://links.lww.com/MSS/D226, for CONSORT Flow Diagram). All participants were considered recreationally active (exercising 4 ± 1 times per week for a total duration of 6 ± 4 h) and were familiar with resistance-type exercise, but none were participating in structured resistance-type exercise training. Participants’ characteristics are presented in Table 1. Participants had no prior history of participating in stable isotope amino acid tracer experiments and were deemed healthy based on their responses to a medical questionnaire. Sample size was initially calculated based on an expected difference in microvascular blood flow of 50% (with a 20% SD) between the intervention and the control condition (12). However, this resulted in a sample size of only four participants, which would have been too low to have enough statistical power for our key secondary outcome, i.e., amino acid incorporation into muscle tissue protein. Therefore, sample size was calculated based on an expected difference of 0.0072 ± 0.006 mole percent excess between the intervention and the control condition (11). The sample size was calculated with a power of 80% and a type I error probability of 0.05. Taking into consideration a dropout rate of 10%, the final number of participants required was 13.

**TABLE 1:** Participants’ characteristics.

	Participants (*n* = 12)
Age (yr)	24 ± 4
Body mass (kg)	80.9 ± 6.9
Height (cm)	183 ± 7
BMI (kg⋅m^−2^)	24.2 ± 2.3
Fat-free mass (kg)	71.3 ± 4.8
Whole body fat mass (kg)	10.0 ± 2.2
Leg press estimated 1RM (kg)	230 ± 31
Leg extension estimated 1RM (kg)	134 ± 22
COLD leg volume (L)	10.8 ± 1.4
CON leg volume (L)	10.6 ± 1.3
COLD leg *m. vastus lateralis* CSA (cm^2^)	24.6 ± 4.6
CON leg *m. vastus lateralis* CSA (cm^2^)	25.0 ± 4.4
COLD leg subcutaneous fat thickness (mm)	22 ± 7
CON leg subcutaneous fat thickness (mm)	23 ± 11

Values are expressed as mean ± SD. COLD, cold-water immersion (8°C); CON, thermoneutral water immersion (30°C); CSA, cross-sectional area.

### Pretesting

All participants participated in a screening session, which was performed at least 1 wk before the start of the experiment. First, participants’ body mass and height were measured, and body composition was assessed by bioelectric impedance (five-segment protocol; BioScan 920-2, Maltron International, Essex, UK). Next, leg volume was assessed based on circumferences at seven sites on both legs. Anthropometric tape was used to measure the circumference at the gluteal furrow, mid-thigh, above the knee, maximum knee, below the knee, as well as maximum calf and minimum ankle circumferences. Positions were marked with a permanent marker for site identification to assess height between circumferences. Leg volume was calculated according to the method described by Jones and Pearson (29). In addition, participants were familiarized with the exercise equipment and performed maximum strength tests as determined by their one-repetition maximum (1RM) for leg press and knee extension exercise using the multiple repetitions testing procedure (30). Participants first performed a 5-min cycling exercise warm-up at 100 W. Thereafter, for both leg press and extension, they performed 2 sets with 10 submaximal or warm-up repetitions to become familiarized with the equipment and to have lifting technique critiqued and corrected. Participants then performed sets at progressively increasing loads until failing to complete a valid repetition, judged by their inability to complete the full range of motion for an exercise. A 2-min resting period between subsequent attempts was allowed. Finally, participants were familiarized with the water immersion procedure. One leg was immersed in cold water (8°C: COLD), while the other leg was immersed in thermoneutral water (30°C: CON) for a total duration of 20 min. Water temperatures between 24°C and 36°C are considered thermoneutral (8). Previous work from our laboratory has shown that both skin and skeletal muscle temperature do not change during and after 20 min of postexercise immersion in 30°C water (11). Furthermore, 20 min of immersion in 8°C water has been used previously as a strong cooling stimulus, while still being practically viable (8,11–14). Both legs were immersed to the level of the gluteal fold. The COLD leg on the experimental test day was randomized between participants’ dominant and nondominant leg (using www.randomizer.org), while the contralateral leg was immersed in thermoneutral water. For the water immersion setup, two water tanks were used that were completely open at the top and contained a tap at the bottom. This allowed us to set and maintain water temperature (before the 20 min water immersion procedure) by adding water and/or ice from the top and remove water from below the tank. During immersion, water was kept still, and temperature was monitored and kept constant at 8°C in COLD and 30°C in CON.

### Diet and physical activity control

All participants received the same standardized dinner (1544 kJ, consisting of 23.4 g protein, 38.7 g carbohydrate, and 11.3 g fat) the evening before the experimental test day. All volunteers refrained from alcohol and any sort of additional exhaustive physical labor and/or exercise 2 d before the experimental test day. To assess compliance, participants filled in food intake and physical activity questionnaires for 2 d before the experimental test day.

### Experimental test day

**FIGURE 1:**
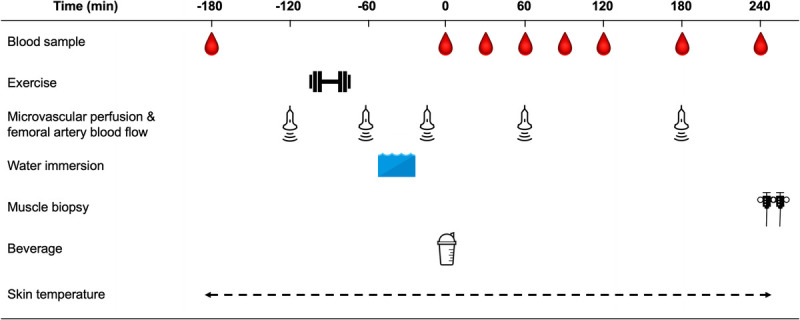
Schematic overview of the experimental test day. Participants performed a single bout of resistance exercise followed by water immersion of both legs (one leg was immersed in 8°C, COLD; the other leg was immersed in 30°C, CON) for 20 min. A recovery beverage consisting of 45 g carbohydrate with a mixture of 20 g free amino acids enriched with L-[ring-^13^C_6_]-phenylalanine was ingested after water immersion. Muscle biopsies were collected from both legs at *t* = 240 min to determine amino acid tracer incorporation. Blood samples were collected, and muscle microvascular perfusion, femoral artery blood flow, and skin temperature measurements were performed throughout the experimental test day.

The outline of the experimental test day is presented in Figure 1. Participants reported to the laboratory in the morning after an overnight fast. First, ibuttons (Maxim Integrated Products) were attached to the skin on the left and right upper thigh (~10 cm above the patella) for continuous measurements of skin temperature during the entire test day. Thereafter, a Teflon catheter was inserted into an antecubital vein for later contrast agent infusion, and a second catheter was inserted in a dorsal hand vein of the contralateral arm, which was subsequently placed in a hotbox (60°C) for “arterialized” blood sampling. After baseline blood sample collection (*t* = −180 min), cross-sectional area of the *m. vastus lateralis* and subcutaneous fat thickness of both legs were assessed at one-third distance between the superior patellar border and the anterior superior iliac spine using ultrasound (Affinity 70G; using the panoramic option with a linear array probe: eL18–4, Philips, the Netherlands). Ultrasound images were later analyzed by manual tracing using ImageJ software (version 2.0.0; National Institutes of Health, Bethesda, MD). Next, resting *m. vastus lateralis* microvascular perfusion was assessed using CEUS (“Baseline”). All CEUS measurements were performed on both legs at the same time using two high-end ultrasound machines (Affinity 70G, Philips, the Netherlands) with linear array probes (eL18-4, Philips, the Netherlands). The ultrasound probe was fixed in a custom-made holder to visualize a cross-sectional image of the *m. vastus lateralis* at one-third distance between the superior patellar border and the anterior superior iliac spine. This position was marked on the leg by pen, and a screenshot in ultrasound B-mode was taken to ensure repeated-measurements at the exact same position. A 5-s clip was acquired in Color Doppler mode to be used during subsequent analysis. Next, an infusion of gas-filled microbubbles (SonoVue, Bracco, concentration: 8μL⋅mL^−1^) was initiated. For each CEUS measurement, a 10-mL suspension of microbubbles was infused for 6 min at 85 mL⋅h^−1^. After 3 min of infusion to achieve a steady state of circulating microbubbles, six 30-s recordings were acquired using contrast mode (8 Hz with a mechanical index [MI] of 0.07). At the start of each recording, a high MI flash (0.53 MI) was given to destroy all visible microbubbles, and the subsequent replenishment of microbubbles was recorded. Immediately after every CEUS measurement, the mean blood velocity and the arterial lumen diameter in the common femoral artery were assessed using the same ultrasound machines and probes in both legs at the same time. First, Doppler ultrasound was used to assess the arterial lumen diameter by video calipers, the measurement was defined as the maximum distance between the media–adventitia interface of the near wall and the lumen–intima interface of the far wall of the vessel. Measurements were made 2–3 cm proximal to the bifurcation of the femoral artery to minimize the effect of turbulence; the insonation angle was <60°. To assess mean femoral artery blood velocity, 30-s video clips were recorded, and mean blood velocity was assessed for each clip using automatic tracing software. Next, femoral artery blood flow (L·min^−1^) was calculated using the system’s software (formula: blood flow = ((*π*) × (femoral artery radius)^2^ × (mean blood flow velocity) × (60))). At every time point that femoral artery blood flow was assessed, systolic and diastolic blood pressure and heart rate were determined as the average of three consecutive measurements (HEM-907, OMRON Healthcare Europe B.V., Hoofddorp, the Netherlands). Subsequently, the participants performed a resistance-type exercise session. After a 5-min warm-up on a cycle ergometer at 100 W, the subjects performed 4 sets of 10 repetitions (at 80% 1RM) on both the leg press and the knee extension exercise machines. After completion of the exercise bout, measurements of *m. vastus lateralis* microvascular perfusion and femoral artery blood flow were repeated (“Post-ex”). Participants then immersed both legs in water for a total duration of 20 min with one leg in cold water (8°C: COLD) and the other leg in thermoneutral water (30°C: CON). Both researchers as well as subjects were not able to be blinded for cooling versus control condition. Immediately after water immersion, measurements of *m. vastus lateralis* microvascular perfusion and femoral artery blood flow were repeated (“Post-im”). For the CEUS measurements after exercise/immersion, the microbubble infusion was started as soon as possible after cessation (approximately 2 min), resulting in the first CEUS measurement at ~5 min after exercise/immersion. Next, a blood sample was taken, and participants subsequently ingested a recovery beverage (see description in next section) at *t* = 0 min. Thereafter, repeated blood samples (*t* = 30, 60, 90, 120, 180, and 240 min) were obtained together with measurements of *m. vastus lateralis* microvascular perfusion and femoral artery blood flow at *t* = 60 min and *t* = 180 min. Finally, muscle biopsies were collected from both legs at *t* = 240 min from the middle region of the *m. vastus lateralis* (~15 cm above the patella) and 3 cm below entry through the fascia, by using the percutaneous needle biopsy technique custom-adapted for manual suction (31). The order of muscle biopsy collection from the legs (first COLD leg or first CON leg) was randomized. All biopsy samples were freed from any visible adipose tissue and blood and immediately frozen in liquid nitrogen and stored at −80°C until subsequent analysis.

### Recovery beverage

Participants ingested a total beverage volume of 500 mL. The beverage contained 45 g of carbohydrate (Maltodextrin, AVEBE MD 14 P, Royal Avebe Veendam), 20 g free amino acid mixture identical to the amino acid composition of milk protein (Ajinomoto, USA, Supplemental Table 1, http://links.lww.com/MSS/D226), and 0.25 g L-[ring-^13^C_6_]-phenylalanine. This was mixed in a bottle up to a total volume of 500 mL with water.

### Microvascular perfusion analysis

All CEUS recordings were analyzed using ImageJ (version 2.1.0/1.53c), as described previously (15). First, the Color Doppler clip was used to create a region of interest (ROI) that excludes larger blood vessels and connective tissue. This ROI was then used for the 30-s microbubble replenishment recording to determine video intensity for every frame. A background correction was applied by subtracting the average video intensity of the first four frames (i.e., 0.5 s) after the high MI flash from all datapoints. Video intensity data were plotted in GraphPad Prism (version 8.3), and a curve was fitted to the equation: *y* = *A*[1 − e^−*βt*^], where *A* is the plateau video intensity (i.e., microvascular blood volume) and *β* reflects the rate of rise of video intensity (i.e., microvascular blood velocity) (32). Microvascular blood flow was calculated as the product of volume and velocity. For one CEUS measurement, the average of the six 30-s recordings was taken for microvascular blood volume, velocity, and flow. All microvascular perfusion analyses were completed in a blinded fashion.

### Plasma analysis

Blood samples (10 mL) were collected in EDTA-containing tubes and centrifuged at 1000*g* and 4°C for 10 min. Aliquots of plasma were frozen in liquid nitrogen and stored at −80°C until analysis. Plasma glucose and insulin concentrations were analyzed using commercially available kits (ref. no. A11A01667, Glucose HK CP; ABX Diagnostics, Montpellier, France; and ref. no. HI-14 K; Millipore, Billerica, MA, respectively). Plasma amino acid concentrations and L-[ring-^13^C_6_]-phenylalanine enrichment were determined by ultra-performance liquid chromatography-mass spectrometry (UPLC-MS; ACQUITY UPLC H-Class with QDa; Waters, Saint-Quentin, France). Specifically, 50 μL blood plasma was deproteinized using 100 μL of 10% SSA with 50 μM of MSK-A2 internal standard (Cambridge Isotope Laboratories, MA). Subsequently, 50 μL of ultra-pure demineralized water was added, and samples were centrifuged (15 min at 21,000*g*). After centrifugation, 10 μL of supernatant was added to 70 μL of Borate reaction buffer (Waters, Saint-Quentin, France). In addition, 20 μL of AccQ/Tag derivatizing reagent solution (Waters, Saint-Quentin, France) was added after which the solution was heated to 55°C for 10 min. Of this, 100 μL derivative 1 μL was injected and measured using UPLC-MS. For the determination of plasma L-[ring-^13^C_6_]-phenylalanine enrichments, phenylalanine was derivatized to its 6-aminoquinolyl-N-hydroxysuccinimidyl carbamate (AQC) derivative, and enrichments were determined by UPLC-MS by using mass detection of masses 336, 342, and 346 for unlabeled and labeled ^13^C_6_ and ^13^C_9 ph_enylalanine, respectively. Standard regression curves were applied from a series of known standard enrichment values against the measured values to assess the linearity of the mass spectrometer and to account for any isotope fractionation, which may have occurred during the analysis. To be used for determination of mixed-muscle protein-bound phenylalanine, plasma protein was extracted from basal blood samples (*t* = −180 min) by adding 20% perchloric acid (PCA) to a final concentration of 2%. Samples were centrifuged at 1000*g* at 4°C for 20 min after which the supernatant was removed. The mixed plasma protein pellet was washed with 3 washes of 2% PCA, after which the supernatant was removed. The protein pellet was hydrolyzed after adding 6 M HCl by heating at 120°C for 15–18 h. The hydrolyzed protein fraction was then dried under a nitrogen stream while being heated to 120°C. Thereafter, the hydrolyzed mixed plasma protein samples were processed via the same procedures as the muscle tissue analyses.

### Muscle analysis

A piece of wet muscle (~50–70 mg) was freeze dried for 48 h. Collagen, excessive blood, and other nonmuscle materials were subsequently removed from the muscle fibers under a light microscope. The isolated muscle fiber mass was weighed, and 35 volumes (7× wet weight of isolated muscle fibers × wet-to-dry ratio 5:1) of ice-cold 2% perchloric acid was added. Thereafter, the tissue was homogenized by sonification and centrifuged to separate the supernatant from the protein pellet. The protein pellet was washed three times with 1 mL 2% PCA. The amino acids were liberated from the mixed-muscle enriched protein fraction by adding 3 mL of 6 M HCl and heating to 110°C for 16 h. The hydrolyzed mixed-muscle protein fractions were dried under a nitrogen stream while heated to 110°C. The dried mixed-muscle protein fraction was dissolved in a 50% acetic acid solution. The amino acids from the mixed-muscle protein fraction were passed over a Dowex exchange resin (AG 50 W-X8, 100–200 mesh hydrogen form; Bio-Rad, Hercules, CA) using 2 M NH4OH. Subsequently, the purified amino acid solution was dried under a nitrogen stream at room temperature, followed by derivatization to their N(O,S)-ethoxycarbonyl-ethyl esters. The ratio of ^13^C/^12^C of mixed-muscle protein-bound phenylalanine was determined using gas chromatography–combustion–isotope ratio mass spectrometry (GC-C-IRMS; MAT253, Thermo Scientific, Bremen, Germany) by monitoring ion masses 44, 45, and 46. Standard regression curves were applied from a series of known standard enrichment values against the measured values to assess the linearity of the mass spectrometer and to account for any isotope fractionation which may have occurred during the analysis.

### Statistical analysis

All data are expressed as mean ± SD. Changes from baseline to postexercise (i.e., “exercise effect”) were analyzed using a two-factor repeated-measures ANOVA with time (Baseline and Post-ex) and treatment (COLD vs CON) as within-subject factors for the analysis of *m. vastus lateralis* microvascular perfusion and femoral artery blood flow parameters. Differences between the legs after the water immersion (i.e., “cooling effect”) were analyzed using a two-factor repeated-measures ANOVA with time (Post-im, *t* = 60, and *t* = 180 min) and treatment (COLD vs CON) as within-subject factors for the analysis of *m. vastus lateralis* microvascular perfusion and femoral artery blood flow parameters. Skin temperature differences between the legs during and after the water immersion were analyzed using a two-factor repeated-measures ANOVA with time (*t* = −50, −45, 40, 35, 30, 20, 10, 0, 20, 40, 60, 120, 180, and 240 min) and treatment (COLD vs CON) as within-subject factors. In case of a significant interaction effect, Student’s paired *t*-tests were used to locate differences between the legs. A Student’s paired *t*-test was used for analysis of amino acid tracer incorporation into muscle tissue. A one-factor repeated-measures ANOVA was performed to analyze changes over time for heart rate and blood pressure. In addition, a one-factor repeated-measures ANOVA was performed to compare plasma glucose, insulin, and amino acid (tracer) concentrations at *t* = 0 min with all other time points (i.e., *t* = 30, 60, 90, 120, 180, and 240 min). Pearson (*r*) correlation analyses were performed between the relative difference between the COLD and the CON legs for amino acid tracer incorporation into muscle tissue and area under the curve from Post-im to *t* = 180 min for femoral artery diameter, blood velocity, blood flow, and microvascular blood volume, velocity, and flow. A significant *r* value between 0 and 0.19 was regarded as “very weak,” between 0.20 and 0.39 as “weak,” between 0.40 and 0.59 as “moderate,” between 0.60 and 0.79 as “strong,” and between 0.80 and 1 as “very strong” correlation. All reported *P* values were adjusted using the Bonferroni–Holm method to correct for multiple comparisons (33). Statistical significance was accepted as *P* < 0.05 (34), and all calculations were performed using SPSS (version 25.0; IBM Corporation).

## RESULTS

### Heart rate and blood pressure

No changes over time were observed for both systolic and diastolic blood pressure (Supplemental Table 2, http://links.lww.com/MSS/D226). Heart rate increased from baseline to postexercise and remained elevated during the postexercise recovery period (Supplemental Table 2, http://links.lww.com/MSS/D226).

### Skin temperature

**FIGURE 2:**
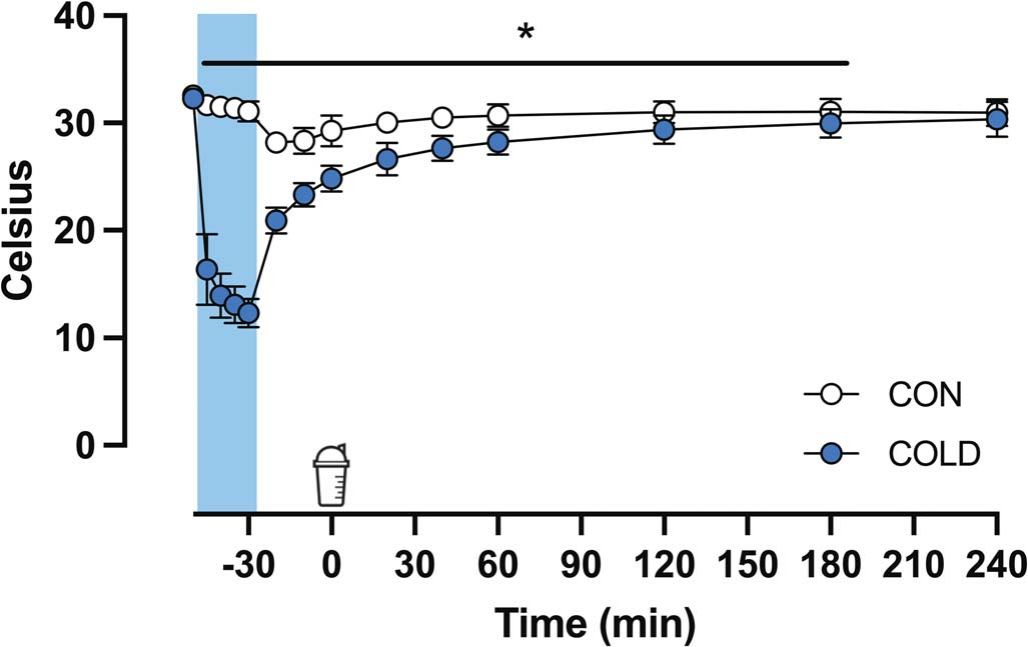
Skin temperature during and after 20 min of cold (8°C: COLD) or thermoneutral water (30°C: CON) immersion in recreationally active young adult males (*n* = 12). The *blue rectangle* represents water immersion from *t* = −50 to *t* = −30 min. The shaker icon represents beverage ingestion at *t* = 0 min. Data were analyzed with the use of two-factor repeated-measures ANOVA. * Significant difference between the COLD and the CON legs, *P* < 0.05.

A significant time*–*treatment interaction was observed for skin temperature (*P* < 0.001, Fig. 2). Skin temperature was lower in the COLD leg compared with the CON leg during water immersion (*t* = −45) until *t* = 180 min (all *P* < 0.01). At *t* = 240 min, the skin temperature of the COLD leg tended (*P* = 0.086) to be lower compared with the CON leg.

### Femoral artery diameter, blood velocity, and blood flow

**FIGURE 3:**
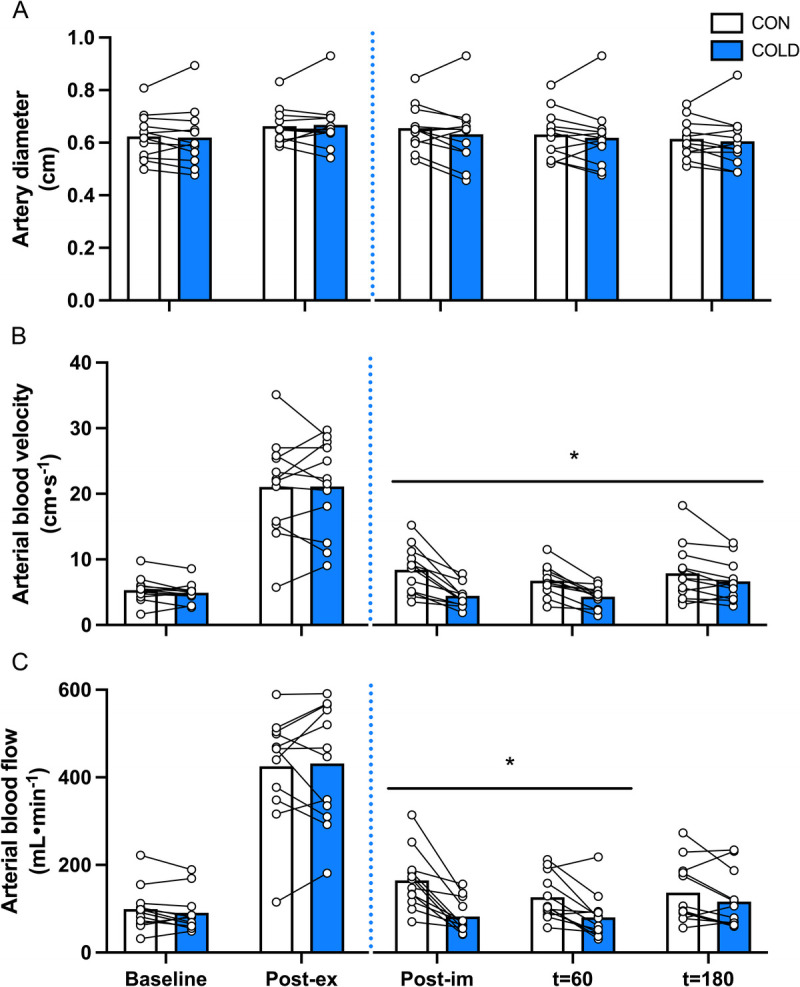
Femoral artery diameter (A), blood velocity (B), and blood flow (C) during the experimental test day in the COLD and CON leg in recreationally active young adult males (*n* = 12). The *blue dotted* line represents water immersion. COLD, cold-water immersion (8°C); CON, thermoneutral water immersion (30°C); Post-ex, postexercise; Post-im, postimmersion; Data were analyzed with the use of two-factor repeated-measures ANOVA. * Significant difference between the COLD and the CON legs, *P* < 0.05.

*Exercise effect:* femoral artery diameter (*P* = 0.001, Fig. 3A), blood velocity (*P* < 0.001, Fig. 3B), and blood flow (*P* < 0.001, Fig. 3C) increased from baseline to postexercise with no differences between the legs.

*Cooling effect:* no significant time–treatment interaction (*P* = 0.39) or main effect of treatment (*P* = 0.31) was observed for femoral artery diameter (Fig. 3A). A significant time–treatment interaction was observed for femoral artery blood velocity (*P* = 0.004, Fig. 3B). Femoral artery blood velocity was lower in the COLD leg compared with the CON leg immediately after water immersion (*P* < 0.001), at *t* = 60 (*P* < 0.001) and *t* = 180 min (*P* = 0.04). A significant time–treatment interaction was also observed for femoral artery blood flow (*P* = 0.001, Fig. 3C). Femoral artery blood flow was significantly lower in the COLD leg compared with the CON leg immediately after water immersion (*P* < 0.001) and at *t* = 60 (*P* = 0.008) and tended to be lower at *t* = 180 min (*P* = 0.091).

### Muscle microvascular blood volume, velocity, and flow

**FIGURE 4:**
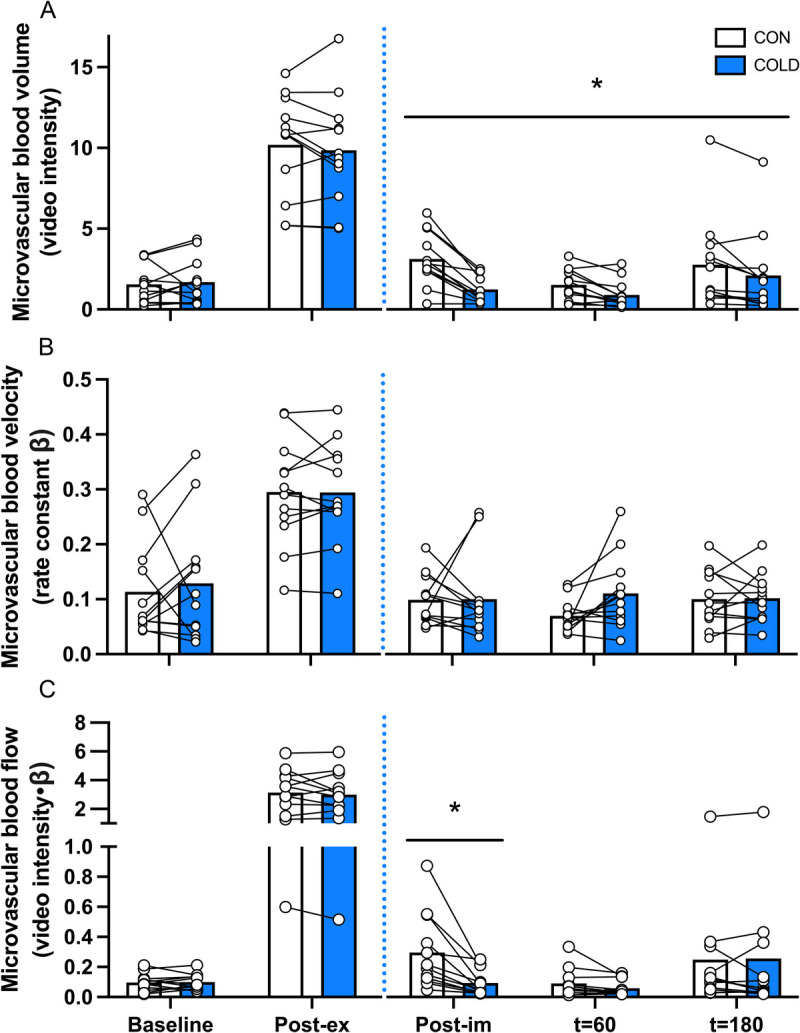
Muscle microvascular blood volume (A), velocity (B), and flow (C), assessed by CEUS, during the experimental test day in the COLD and CON leg in recreationally active young adult males (*n* = 12). The *blue dotted* line represents water immersion. COLD, cold-water immersion (8°C); CON, thermoneutral water immersion (30°C); Post-ex, postexercise; Post-im, postimmersion. Data were analyzed with the use of two-factor repeated-measures ANOVA. * Significant difference between the COLD and the CON legs, *P* < 0.05.

*Exercise effect:* microvascular blood volume (*P* < 0.001, Fig. 4A), velocity (*P* = 0.002, Fig. 4B), and flow (*P* < 0.001, Fig. 4C) increased from baseline to postexercise with no differences between the legs.

*Cooling effect:* a significant time–treatment interaction was observed for microvascular blood volume (*P* < 0.001, Fig. 4A). Microvascular blood volume was lower in the COLD leg compared with the CON leg immediately after water immersion (*P* < 0.001), at *t* = 60 (*P* = 0.016) and *t* = 180 min (*P* = 0.027). No significant time–treatment interaction (*P* = 0.29) or main effect of treatment (*P* = 0.74) was observed for microvascular blood velocity (Fig. 4B). A significant time–treatment interaction was observed for microvascular blood flow (*P* = 0.035, Fig. 4C). Microvascular blood flow was lower in the COLD leg compared with the CON leg immediately after water immersion (*P* = 0.015). No differences between the COLD and CON legs were observed for microvascular blood flow at *t* = 60 (*P* = 0.14) or *t* = 180 min (*P* = 0.84).

### Plasma concentrations

**FIGURE 5:**
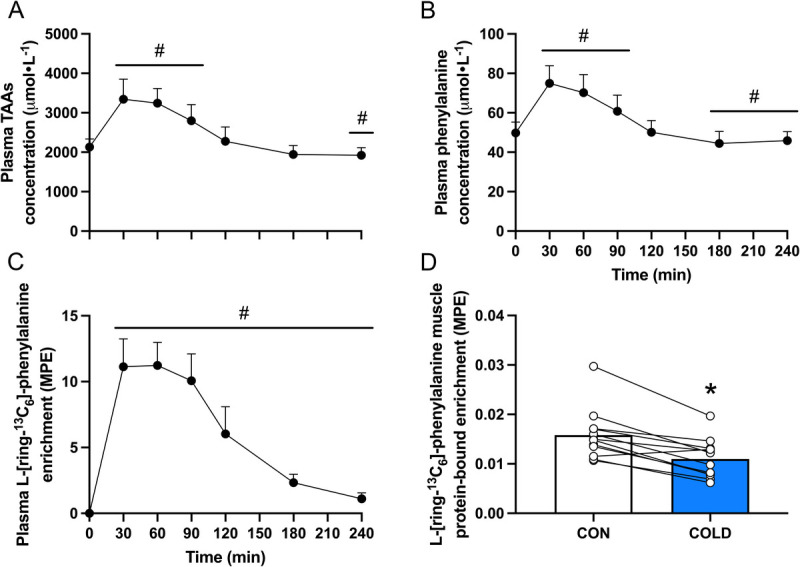
Plasma total amino acid (TAA, A), phenylalanine (B) concentration, L-[ring-^13^C_6_]-phenylalanine enrichment (C), and L-[ring-^13^C_6_]-phenylalanine muscle protein-bound enrichment (D) after beverage ingestion (*t* = 0) in recreationally active young adult males (*n* = 12). MPE, mole percent excess; COLD, cold-water immersion (8°C); CON, thermoneutral water immersion (30°C). Plasma data were analyzed with the use of one-factor repeated-measures ANOVA. Amino acid tracer incorporation into muscle tissue was analyzed with the use of a Student’s paired *t*-test. # Significantly different from *t* = 0 min, *P* < 0.05. * Significantly different from CON, *P* < 0.05.

Plasma total amino acids concentration increased above baseline (*t* = 0 min) at *t* = 30, *t* = 60, and *t* = 90 min (all *P* < 0.001) before returning to baseline at *t* = 120 min, and then reaching values slightly lower than baseline at *t* = 180 (*P* = 0.052) and *t* = 240 min (*P* = 0.015, Fig. 5A). Similarly, plasma phenylalanine concentration increased above baseline at *t* = 30 (*P* < 0.001), *t* = 60 (*P* < 0.001), and *t* = 90 min (*P* = 0.004) and was lower than baseline at *t* = 180 (*P* = 0.015) and *t* = 240 min (*P* = 0.034, Fig. 5B). Similar patterns were observed for plasma leucine, branched-chain amino acid, essential amino acid, and nonessential amino acid concentrations (see Supplemental Fig. 2, http://links.lww.com/MSS/D226). Plasma L-[ring-^13^C_6_]-phenylalanine enrichment was increased above baseline (*t* = 0 min) at all other time points (all *P* < 0.001, Fig. 5C). Plasma glucose concentration was increased above baseline at *t* = 30 (*P* < 0.001) and *t* = 60 (*P* = 0.012), but below baseline at *t* = 120 (*P* = 0.024), *t* = 180 (*P* = 0.005), and *t* = 240 min (*P* = 0.018, Table 2). Plasma insulin concentration increased above baseline (*t* = 0 min) at *t* = 30 (*P* < 0.001), *t* = 60 (*P* = 0.004), and *t* = 90 min (*P* = 0.005, Table 2).

**TABLE 2:** Plasma glucose and insulin concentrations after beverage ingestion (*t* = 0) in recreationally active young adult males (*n* = 12).

Time (min)	0	30	60	90	120	180	240
Glucose concentrations (mmol⋅L^−1^)	5.0 ± 0.3	7.6 ± 1.1*	6.0 ± 0.7*	4.9 ± 0.8	4.6 ± 0.5*	4.6 ± 0.3*	4.7 ± 0.2*
Insulin concentrations (pg⋅mL^−1^)	286 ± 296	4679 ± 2846*	2113 ± 1257*	684 ± 470*	226 ± 169	134 ± 60	140 ± 75

Values are expressed as mean ± SD. Data were analyzed with the use of one-factor repeated-measures ANOVA.

* Significantly different from *t* = 0, *P* < 0.05.

### Amino acid tracer incorporation into muscle tissue

L-[ring-^13^C_6_]-phenylalanine mixed-muscle protein-bound enrichment at *t* = 240 min after beverage ingestion was lower in the COLD leg compared with the CON leg (0.011 ± 0.004 vs 0.016 ± 0.005 mole percent excess, respectively, *P* < 0.001, Fig. 5D).

### Correlations

**FIGURE 6:**
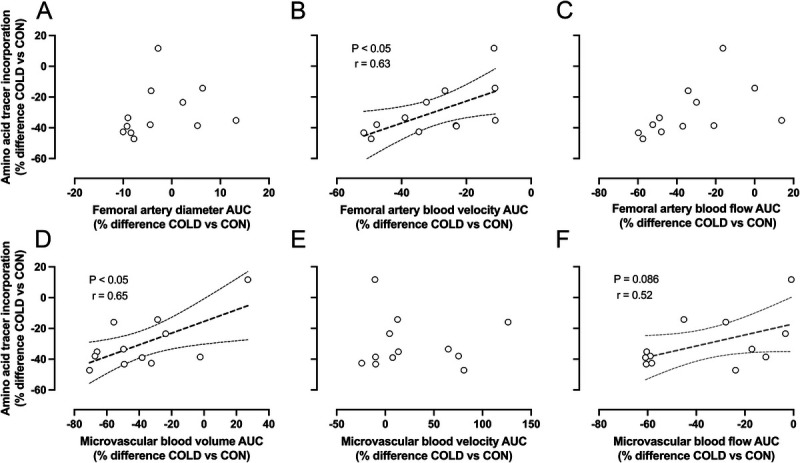
Pearson correlations (*r*) between amino acid tracer incorporation into mixed-muscle protein and femoral artery diameter (A), blood velocity (B), blood flow (C), microvascular blood volume (D), velocity (E), and flow (F) in recreationally active young adult males (*n* = 12). Straight lines represent linear regression and bands represent the 95% confidence interval. COLD, cold-water immersion (8°C); CON, thermoneutral water immersion (30°C); AUC, area under the curve; calculated using the postimmersion, *t* = 60 and *t* = 180 min time points. All values used for correlated analyses were expressed as % difference between the COLD and the CON legs.

No significant correlations were observed between the difference in amino acid tracer incorporation into muscle tissue and the difference in femoral artery diameter (Fig. 6A) or blood flow (Fig. 6C) between the COLD and the CON legs. By contrast, a strong significant correlation was observed between the difference in amino acid tracer incorporation into muscle tissue and the difference in femoral artery blood velocity between both legs (Fig. 6B). No significant correlation was observed between the difference in amino acid tracer incorporation into muscle tissue and the difference in microvascular blood velocity between both legs (Fig. 6E). However, moderate to strong significant correlations were observed between the difference in amino acid tracer incorporation into muscle tissue and the difference in microvascular blood volume (Fig. 6D) and flow (Fig. 6F) between the COLD and the CON legs.

## DISCUSSION

The present study shows that cold-water immersion during recovery from resistance exercise greatly reduces muscle microvascular blood volume in recreationally active young adult males. This reduction was strongly related to a lower postprandial amino acid incorporation into muscle tissue in the cooled versus thermoneutral leg during postexercise recovery.

The increase in arterial blood flow after exercise is important for adequate blood supply toward muscle tissue to support recovery (35). In this study, we show that a bout of bilateral lower-body resistance exercise substantially increased femoral artery blood flow (+350%), assessed by Doppler ultrasound. After exercise cessation, one leg was submerged in cold water (8°C), whereas the other leg was submerged in thermoneutral water (30°C), for a 20-min duration followed by ingestion of a postexercise recovery drink. Postexercise cooling resulted in a ~20°C drop in skin temperature, which is in line with our previous work (11). Although this does not reflect the change in thigh muscle temperature *per se*, we have previously shown that this magnitude in skin temperature decline relates to a ~5°C lower muscle temperature in a cooled compared with a control leg (11). Immediately after completing the water immersion protocol and at multiple times after drink ingestion, femoral artery blood flow was assessed. Femoral artery blood flow was observed to be ~50% lower in the COLD compared with the CON leg after water immersion, which is in line with previous studies (12,13). The observed reduction in arterial blood flow was mainly driven by a decrease in blood velocity, as artery diameter was not different between the COLD and the CON legs. Femoral artery blood velocity remained lower (16%) in the COLD compared with the CON leg, even at *t* = 180 min after drink ingestion. Although cold-water immersion clearly has a profound negative impact on the blood supply toward the lower limb during postexercise recovery, this does not necessarily reflect a change in skeletal muscle tissue perfusion as it also includes blood flow toward the skin (12,13).

Perfusion of skeletal muscle tissue is of key importance to support postexercise recovery and adaptation as it provides oxygen, nutrients, and growth factors to the muscle fibers (16). In the present study, we used CEUS in a within-subject design to assess *vastus lateralis* muscle microvascular perfusion at multiple time points after exercise and water immersion in both legs simultaneously. By excluding larger blood vessels (i.e., arteries and veins) from a set ROI within a specified muscle section, CEUS can be applied to assess microvascular perfusion (18–22). Microvascular blood flow (i.e., the product of microvascular blood volume and velocity) was greatly increased nearly ~30-fold after exercise in both legs. Previous work has suggested that cold-water immersion could lower subsequent muscle microvascular perfusion during postexercise recovery (14). Here, we present the first study to directly assess muscle microvascular perfusion after cold-water immersion during postexercise recovery. Immediately after water immersion, microvascular blood flow was on average 68% lower in the COLD leg when compared with the thermoneutral CON leg. Interestingly, the observed reduction in microvascular blood flow in the COLD leg was mainly driven by changes in microvascular blood volume, not velocity. Although microvascular blood volume remained lower (24%) until *t* = 180 min after beverage ingestion in the COLD leg, microvascular blood velocity was unaffected by cooling. These results clearly show that cold-water immersion has a negative impact on muscle microvascular perfusion and may, therefore, compromise nutrient delivery during postexercise recovery (36).

Postexercise protein administration is required to provide amino acids to augment the exercise induced increase in muscle protein synthesis and, as such, support muscle tissue conditioning (23–27). After ingestion, amino acids are absorbed and released into the circulation and subsequently delivered to the muscle fibers via the microvasculature (28). In this study, participants ingested 45 g of carbohydrate with a mixture of 20 g free amino acids enriched with L-[ring-^13^C_6_]-phenylalanine after postexercise water immersion. After ingestion of the recovery beverage, we observed a rapid rise in circulating amino acid concentrations with a concomitant rise in L-[ring-^13^C_6_]-phenylalanine enrichment (Figs. 5A-C). At *t* = 240 min after beverage ingestion, a muscle biopsy was taken from both legs to assess amino acid tracer incorporation into muscle tissue. The L-[ring-^13^C_6_]-phenylalanine incorporation into mixed-muscle protein was ~30% lower in the COLD compared with the CON leg (Fig. 5D). These differences in *de novo* muscle protein synthesis seem to be secondary to differences in muscle tissue perfusion, as we observed a strong correlation between exogenous amino acid incorporation and microvascular blood volume in the COLD and CON leg. This suggests that the cold-water immersion induced reduction in muscle microvascular perfusion compromises postexercise nutrient delivery and attenuates the postexercise increase in muscle protein synthesis.

Cold-water immersion is often applied by both recreational and professional athletes to improve postexercise recovery and thereby “optimize” the muscle reconditioning response (1). In support, postexercise cooling may provide some (psychological) benefits during postexercise recovery (2–9). However, although not assessed in the current study, we have previously shown a reduction in postexercise muscle protein synthesis rates when cold-water immersion is applied during postexercise recovery (11). In the same study, the reduction in postexercise muscle protein synthesis rates could not be explained by differences in anabolic signaling, and it was speculated that a cold-induced decrease in muscle tissue perfusion may play an important role (11). The present study extends on these findings by showing that postexercise cold-water immersion lowers muscle microvascular perfusion up to ~4 h after exercise cessation. This reduced blood flow is strongly associated with a reduced exogeneous amino acid incorporation into muscle tissue when a recovery beverage is ingested after exercise. It should be noted that only males were assessed in the current study. It is possible that the observed effects may be smaller in populations with a thicker upper thigh subcutaneous fat layer, which is more likely observed in females and older adults. In these populations, the drop in muscle temperature could be less pronounced due to greater insulation by subcutaneous fat (37). Furthermore, based on the present observations, it can be speculated that postexercise muscle tissue heating may have the opposite effect, i.e., enhancing postexercise microvascular perfusion and nutrient delivery. By contrast, we have previously shown that hot-water immersion does not affect postexercise muscle protein synthesis rates in healthy young males (38). However, it would still be interesting to investigate whether postexercise muscle tissue heating could be beneficial in populations with impaired vascular function and muscle protein turnover, such as healthy or more clinically compromised older adults. Together, the results of the current study contribute to the growing evidence that postexercise cold-water immersion compromises resistance exercise training induced muscle conditioning. Hence, individuals aiming to maximize their skeletal muscle adaptive response to exercise training should reconsider incorporating postexercise cooling in their recovery routine.

In conclusion, cold-water immersion during recovery from resistance exercise greatly reduces microvascular blood volume, which is strongly related to a lower postprandial amino acid incorporation in skeletal muscle protein in recreationally active males. Cold-water immersion should be avoided during postexercise recovery to ensure proper nutrient delivery to muscle tissue to maximize postexercise muscle conditioning.
